# Identification of superior haplotypes in a diverse natural population for breeding desirable plant height in soybean

**DOI:** 10.1007/s00122-022-04120-0

**Published:** 2022-05-31

**Authors:** Javaid Akhter Bhat, Benjamin Karikari, Kehinde Adewole Adeboye, Showkat Ahmad Ganie, Rutwik Barmukh, Dezhou Hu, Rajeev K. Varshney, Deyue Yu

**Affiliations:** 1 State Key Laboratory of Crop Genetics and Germplasm Enhancement, National Center for Soybean Improvement, Nanjing Agricultural University, Nanjing, 210095 China; 2 International Genome Center, Jiangsu University, Zhenjiang, 212013 China; 3 Department of Crop Science, Faculty of Agriculture, Food and Consumer Sciences, University for Development Studies, Tamale, Ghana; 4 Department of Agricultural Technology, Ekiti State Polytechnic, P. M. B. 1101, Isan, Nigeria; 5 Department of Plant Science and Landscape Architecture, University of Connecticut, Storrs, USA; 6 Center of Excellence in Genomics and Systems Biology, International Crops Research Institute for the Semi-Arid Tropics (ICRISAT), Hyderabad, 502324 India; 7 Murdoch’s Centre for Crop and Food Innovation, State Agricultural Biotechnology Centre, Food Futures Institute, Murdoch University, Murdoch, WA Australia

## Abstract

**Key message:**

Plant height of soybean is associated with a haplotype block on chromosome 19, which classified 211 soybean accessions into five distinct groups showing significant differences for the target trait.

**Abstract:**

Genetic variation is pivotal for crop improvement. Natural populations are precious genetic resources. However, efficient strategies for the targeted utilization of these resources for quantitative traits, such as plant height (PH), are scarce. Being an important agronomic trait associated with soybean yield and quality, it is imperative to unravel the genetic mechanisms underlying PH in soybean. Here, a genome-wide association study (GWAS) was performed to identify single nucleotide polymorphisms (SNPs) significantly associated with PH in a natural population of 211 cultivated soybeans, which was genotyped with NJAU 355 K Soy SNP Array and evaluated across six environments. A total of 128 SNPs distributed across 17 chromosomes were found to be significantly associated with PH across six environments and a combined environment. Three significant SNPs were consistently identified in at least three environments on Chr.02 (AX-93958260), Chr.17 (AX-94154834), and Chr.19 (AX-93897200). Genomic regions of ~ 130 kb flanking these three consistent SNPs were considered as stable QTLs, which included 169 genes. Of these, 22 genes (including Dt1) were prioritized and defined as putative candidates controlling PH. The genomic region flanking 12 most significant SNPs was in strong linkage disequilibrium (LD). These SNPs formed a single haplotype block containing five haplotypes for PH, namely Hap-A, Hap-B, Hap-C, Hap-D, and Hap-E. Deployment of such superior haplotypes in breeding programs will enable development of improved soybean varieties with desirable plant height.

**Supplementary Information:**

The online version contains supplementary material available at 10.1007/s00122-022-04120-0.

## Introduction

Cultivated soybean (*Glycine max* L.) is an important oilseed crop, grown globally as a major source of edible protein and oil (Kulkarni et al. 2016). However, in the past five decades, only a gradual increase in soybean yield has been observed in China. This can be witnessed by the fact that China imports > 80% of soybean and its products to meet its domestic requirements (Liu et al. 2018). Hence, development of soybean genotypes with desirable plant architecture and higher yield is a major objective of soybean breeders across the world, and especially from China. A significant positive correlation has been observed between PH and yield in soybean. However, taller soybean genotypes are highly prone to lodging, affecting the overall yield and seed quality (Liu et al. 2011; Lü et al. 2016). This makes PH a crucial trait in the breeding programs of soybean. Moreover, PH is a quantitative trait that is controlled by multiple loci/genes, besides being highly influenced by genotype × environment interactions (Wang et al. 2004; Cao et al. 2019). Over the years, breeding for desirable PH has been accomplished via both traditional and marker-assisted breeding (MAB) approaches (Bhat et al. 2020). In the case of environment-sensitive traits such as PH, the MAB approaches are much more effective and allow selection of plants at initial growth stages, such as germination or seedling, even for traits measured at maturity (Allen 1994; Chapman et al. 2003). Therefore, elucidating the genetic basis of PH is expected to greatly facilitate implementation of MAB in the development of high-yielding soybean varieties.

Some studies have documented the genetic basis of PH in soybean (Mansur et al. 1996; Orf et al. 1999; Liu et al. 2013; Yin et al. 2017). For instance, the past three decades have witnessed identification of > 200 quantitative trait loci (QTLs), as documented in the SoyBase (https://www.soybase.org/), and these QTLs are located across 20 soybean chromosomes. Most of the previously reported studies used linkage mapping with low-resolution markers to identify such QTLs, which often possess low genomic resolution. This has hampered successful deployment of PH QTLs in breeding programs to develop improved soybean varieties with desired PH (Cao et al. 2019). An alternative approach of trait mapping such as GWAS detects alleles/QTLs linked to the trait of interest at a higher resolution using diverse natural populations (Schmutz et al. 2010). Technological advances in next-generation sequencing-based genotyping technologies have enabled GWAS to be used routinely in crop genetics research (Song et al. 2016; Cao et al. 2019). Several studies have demonstrated the efficiency of GWAS to identify marker-trait associations (MTAs) regulating important agronomic traits and to detect the underlying putative genes in different plant species such as *Arabidopsis thaliana* (Ren et al. 2019), rice (Xu and Crouch 2008; Li et al. 2014), maize (Yang and Zhu 2005), wheat (Wang et al. 1999), cotton (Lai et al. 2016), soybean (Hyten et al. 2008; Cao et al. 2017; Fang et al. 2017; Li et al. 2017; Chang et al. 2018), chickpea (Varshney et al. 2019a, b; Thudi et al. 2021), pigeonpea (Varshney et al. 2017a, b), and pearl millet (Varshney et al. 2017a, b).

With the availability of whole genome sequencing data (WGS), sequencing-based trait mapping strategies have been successful in identifying QTLs/ MTAs in several crops (Varshney et al. 2019a, b). Notably, the use of WGS data has increased the mapping resolution of GWAS and enabled identification of haplotypes for the trait(s) of interest. In the post-sequencing era*,* ‘haplotype assembly’ has emerged as an efficient approach for the development of improved crop varieties (Bevan et al. 2017). In the past few years, *haplo-pheno* analysis has been successfully used for detecting superior haplotypes for agronomically important traits in some crop species. For example, Abbai et al. (2019) reported desirable haplotypes for 21 genes controlling quality and yield-related traits in rice, based on a panel of 3000 rice accessions. Similarly, superior haplotypes have been identified for drought and salinity stress adaptation in rice, soybean, and pigeonpea (Guan et al. 2014; Mishra et al. 2016; Kuroha et al. 2018; Chen et al. 2019; Sinha et al. 2020).

To understand the genetics of PH in soybean genotypes selected from major soybean growing areas of China, the present study used GWAS and haplotype analysis for identifying MTAs, candidate genes, and haplotypes. Superior haplotypes identified in this study hold potential for developing improved soybean varieties with desired plant architecture using haplotype-based breeding approach.

## Material and methods

### Plant material and field experiments

A natural population comprising 219 soybean germplasm accessions was evaluated for two consecutive years (2011 and 2012) at three different locations in China. This soybean germplasm represents the cultivated pool selected from different ecological regions of China, previously described by Wang et al. (2016) and Du et al. (2020) (Supplementary Table 1). The evaluated locations include the experimental fields of Nanjing Agricultural University (Nanjing); Jiangsu Yanjiang-Institute of Agricultural Sciences (Nantong); and Agricultural College of Yangzhou University (Yangzhou). Hence, the germplasm was evaluated at a total of six individual environments (location × year), namely E1–E6 (E1: Nanjing_2011, E2: Nantong_2011, E3: Yangzhou_2011, E4: Nanjing_2012, E5: Nantong_2012, and E6: Yangzhou_2012). These three locations differ substantially in temperature, precipitation, and soil quality. For example, Nanjing (32°12′ N, 118°37′ E) has a north subtropical humid climate with four distinct seasons. The average rainfall and relative humidity in Nanjing are 1106.5 mm and 76%, respectively. The annual average temperature in Nanjing is 15.4 °C, and the soil quality of Nanjing is mostly red soil with high iron content. Further, Nantong (31°58′ N, 120°53′ E) is in the alluvial plain at lower reaches of the Yangtze River with mild marine climate. The annual average temperature and precipitation in Nantong are 15.1 °C and ~ 1040 mm, respectively. There are four distinct seasons in Nantong, and the spring and autumn seasons are relatively short. The soil in Nantong is mainly yellow brown. Yangzhou (32°23′ N, 119°25′ E) is located at the southern end of the Yangtze Huaihe plain. It is affected by monsoon circulation, with four distinct seasons and a mild climate. The annual average temperature is 14.8 °C, and the average precipitation is 1020 mm. The soil texture of Yangzhou is mainly heavy soil, and a small part of it is sandy and light clay. The experiments were conducted in a complete randomized block design with three replications of each genotype. The field data for PH collected from six different environments are provided in Supplementary Table 2. Standard soybean agronomic cultural practices (Zhang et al. 2015) were followed at each location during the experiments.

### Phenotypic data collection and statistical analysis

In each environment, five consecutive plants of each genotype were selected from the middle of each plot for PH phenotyping in each replication. PH was measured in centimeters (cm) by using a measuring tape stretched from the cotyledonary node to the top of the plant at the maturity stage. The data obtained from individual environments (location × year combination) and the combined environment were evaluated using “*lme4*” package in R environment. The analysis of variance (ANOVA) and predicted means [best linear unbiased predictions (BLUPs)] were calculated with genotype set as random. Predicted means (BLUPs) for individual environment were estimated using the model:

$$Y_{ij} = \mu + {\text{Rep}}_{i} + {\text{Gen}}_{j} + \varepsilon_{ij}$$

where *Y*_*ijk*_ is the trait (PH), *µ* is the overall mean effect, and Rep_*i*_ denotes the effect of the *i*th replicate/block. Gen_*j*_ represents the effect of the *j*th genotype; *ε*_*ij*_ is the effect of the error associated with the *i*th replication/block, and *j*th genotype.

The ANOVA and predicted means (BLUPs) for the combined environment were estimated using the following model:

$$Y_{ijk} = \mu + {\text{Env}}_{i} + {\text{Rep}}_{j} \left( {{\text{Env}}_{i} } \right) + {\text{Gen}}_{k} + {\text{Env}}_{i} \times {\text{Gen}}_{k} + \varepsilon_{ijk}$$

where Env_*i*_ and Env_*i*_ × Gen_*k*_ are the *i*th environment and the *G* × *E* interaction effects, respectively.

The broad-sense heritability for the combined and individual environment was estimated as *σ*^2^_g_/(*σ*^2^_g_ + *σ*^2^_ge_/*n* + *σ*^2^_ε_/*nr*) and *σ*^2^_g_/(*σ*^2^_g_ + *σ*^2^_ε_/*r*), respectively, where *σ*_g_ is the genetic variance, *σ*^2^_ge_ is the interaction of genotype and environment, *σ*^2^_ε_ is the residual error, *n* is the number of environments, and *r* is the number of replication/block (Knapp et al. 1985).

To identify the relationship between PH evaluated across different environments, we performed a Pearson’s correlation analysis. In brief, Pearson correlation analysis for soybean PH measured across different environment was conducted based on the predicted means calculated across the environment and visualized using MVApp (Julkowska et al. 2019).

### Genome-wide association study

The NJAU 355 K SoySNP assay previously developed by our laboratory (Wang et al. 2016) was used in the present study. In brief, the sequencing data of 31 soybean genotypes (including 14 cultivated and 18 wild type) were used along with Williams 82 (Glyma.Wm82.a1.v1.1) as the reference genome (www.phytozome.net). The VIP variants include 60,800 SNPs from SoySNP50K15, 2103 SNPs from SoyBase, and 3671 SNPs from the sequencing data of 31 soybean genotypes. A total of 355,595 SNPs representing 609,883 probe sets were obtained following the Affymetrix filtering (Wang et al. 2016). The SNP dataset has been provided as Supplementary Table 3. In the current study, for the selection of high-quality SNPs, quality control analysis was conducted at minimum minor allele frequency (MAF) of 0.01 and missing genotype and taxa at 0.2 in TASSEL v5.2.73 (Bradbury et al. 2007).

Genome-wide association analysis was conducted with Genomic Association and Prediction Integrated Tool (GAPIT) package in R environment (Lipka et al. 2012) using a compressed mixed linear model (CMLM). This model was adopted to speed up computational time and optimize statistical performance (Lipka et al. 2012). UpSet plot was developed for significant SNPs by using the *UpSetR* package in R environment (Lax et al. 2014; Conway et al. 2017).

### Haplotype analysis

The LD level of pairwise SNPs was calculated using Haploview 4.2 (Barnett et al. 2005). All markers associated with PH on chromosome 19 (Chr.19) within the LD ± 130 kb (Wang et al. 2016) were considered, and haplotype block was defined by “confidence intervals” algorithm (Gabriel et al. 2002). Genotypes were grouped into independent groups based on specific haplotype carried by them. To estimate the effect of haplotype on PH, groups were fitted into one-way ANOVA model in R environment as follows:

$${\text{model}} \leftarrow {\text{aov}}\left( {{\text{phenotype}}\sim{\text{group}},{\text{ data}} = {\text{data}}} \right)$$

where phenotype denotes PH values in the individual and combined environments. A pairwise comparison of means was conducted using Turkey’s HSD test and visualized in R environment.

### Identification of candidate genes

Within the LD decay distance of significant SNPs, all genes present within the ± 130 kb interval flanking the stable SNP positions were retrieved from SoyBase website (https://www.soybase.org/), using *Glyma2.0* gene model. The functional annotations of genes were downloaded from SoyBase, and were screened manually. The RNA-seq data available at SoyBase (https://www.soybase.org/) were extracted for the genes underlying the QTL intervals from different soybean tissues (leaf, flower, pod shell, seed, root, and nodule) at different growth and developmental stages, viz., young leaf, flower, one cm pod, pod shell 10 days to flowering (DAF), pod shell 14DAF, seed 10DAF, seed 14DAF, seed 21DAF, seed 25DAF, seed 28DAF, seed 35DAF, seed 42DAF, root, and nodule) (Supplementary Table 4). These RNA-seq data were previously generated and deposited in the SoyBase by Severin et al. (2010). The RNA-seq dataset was used to analyze the expression of putative genes in different soybean tissues and across multiple development stages. For developing a heat map, the fragments per kilobase of transcript per million fragment mapped (FPKM) values of the candidate genes were used. Hierarchical clustering was performed using Euclidean Distance Method with complete linkage approach (Tao et al. 2013). A heatmap of these candidate genes was constructed using TBtools_JRE1.6 software (Chen et al. 2020). Based on functional annotations of genes, available literature, and gene expression patterns, some genes were defined as putative candidates regulating PH.

## Results

### Phenotypic characterization and correlation analysis of PH in soybean accessions

The descriptive statistics including mean, range (maximum and minimum values), standard deviation, skewness, kurtosis, broad-sense heritability, and coefficient of variation (CV) for PH in 219 soybean accessions evaluated across six different environments and the combined environment are presented in Table 1. In brief, the PH ranged from 8.24 cm in E6 to 311.67 cm in E4. The mean across individual environments ranged from 52.76 ± 0.83 cm in E3 to 105.30 ± 1.73 in E4. The CV in the combined environment was 49%, and ranged from 38% in E6 to 49.8% in E2. Furthermore, skewness and kurtosis were 1.34 and 2.84, respectively in the combined environment (Table 1). The broad-sense heritability ranged from 0.84 in E6 to 0.98 in E4, and 0.97 in the combined environment. The soybean accessions (*G*), environment (*E*), and the genotype × environment interaction (*G* × *E*) had significant effect (*P* < 0.0001) on PH (Table 2). Moreover, Pearson correlation analysis of PH showed a significantly positive correlation (*P* < *0.0001*) across different environments evaluated (Supplementary Table 5).

**Table 1:** Descriptive statistics, broad-sense heritability, and coefficient of variation (CV) for plant height evaluated across 211 diverse soybean accessions

Environment	Minimum	Maximum	Mean ± SE	SD	CV%	Skewness	Kurtosis	Broad-sense heritability
CE	8.24	311.67	74.68 ± 0.60	36.69	49.13	1.34	2.84	0.97
E1	35.00	243.00	85.83 ± 1.40	33.78	39.36	0.80	0.33	0.95
E2	8.33	199.33	61.86 ± 1.26	30.82	49.83	1.34	2.24	0.94
E3	21.00	155.00	52.76 ± 0.83	21.27	40.31	1.01	1.65	0.97
E4	29.75	311.67	105.30 ± 1.73	44.25	42.02	1.20	2.15	0.98
E5	18.67	191.67	69.61 ± 1.27	32.13	46.15	1.07	0.78	0.95
E6	8.24	212.67	72.51 ± 1.10	27.72	38.23	1.01	1.95	0.84

*SE* Standard error

**Table 2:** Combined analysis of variance (ANOVA) for plant height evaluated across six environments

Source	DF	SS	MS	*F-*Value	Pr*(*> *F)*
Block within *E*	12	47,479.90	3956.66	27.93	
Environment (*E*)	5	1,119,552.13	223,910.43	56.59	< 0.0001
Genotype (*G*)	218	3,085,979.28	14,155.87	99.94	< 0.0001
*G* × *E*	1084	656,054.90	605.22	4.27	< 0.0001
Error	2457	348,007.48	141.64		
Total	3776	5,257,073.68			

*DF* Degrees of freedom; *SS* Sum of squares; *MS* Mean sum of squares; *E* Environment

### Genome-wide distribution of SNPs in soybean natural population

In the present study, a total of 211 soybean accessions and 291,962 SNP markers were retained after quality control analysis (Table 3). These markers covered 949,992,372 bp (949.99 Mb) of the soybean genome, representing ~ 85% of the genome. The number of SNPs in each chromosome varied from 12,035 to 19,880, with the lowest number of SNPs present on Chr.11, whereas Chr.18 contained the highest number of SNPs. The lowest and highest SNP density of 264.27 SNPs/Mb and 361.97 SNPs/Mb was found on Chr.01 and Chr.13, respectively (Supplementary Fig. 1; Table 3).

**Table 3:** Distribution of 291,962 SNPs on 20 soybean chromosomes used for GWAS analysis of plant height in a diverse soybean population

Chromosome	Length (bp)	Length (Mb)	Number of SNPs	Inter-marker distance (kb)	Density (SNPs/Mb)
Chr.01	55,915,249	55.92	14,777	3.8	264.27
Chr.02	51,649,736	51.65	15,318	3.4	296.57
Chr.03	47,780,218	47.78	14,653	3.3	306.68
Chr.04	49,243,023	49.24	13,793	3.6	280.10
Chr.05	41,932,848	41.93	12,557	3.3	299.45
Chr.06	50,704,308	50.70	16,001	3.2	315.57
Chr.07	44,682,118	44.68	13,744	3.3	307.60
Chr.08	46,990,565	46.99	15,346	3.1	326.58
Chr.09	46,842,345	46.84	14,657	3.2	312.90
Chr.10	50,966,905	50.97	14,887	3.4	292.09
Chr.11	39,171,759	39.17	12,035	3.3	307.24
Chr.12	40,112,060	40.11	12,638	3.2	315.07
Chr.13	44,406,433	44.41	16,074	2.8	361.97
Chr.14	49,707,254	49.71	15,350	3.2	308.81
Chr.15	50,937,513	50.94	15,113	3.4	296.70
Chr.16	37,392,142	37.39	12,760	2.9	341.25
Chr.17	41,900,927	41.90	13,387	3.1	319.49
Chr.18	62,305,715	62.31	19,880	3.1	319.07
Chr.19	50,585,940	50.59	15,502	3.3	306.45
Chr.20	46,765,314	46.77	13,490	3.5	288.46
Total	949,992,372	949.99	291,962	65.15	6166.32

### GWAS revealed genetic architecture of PH in soybean

**Fig. 1:**
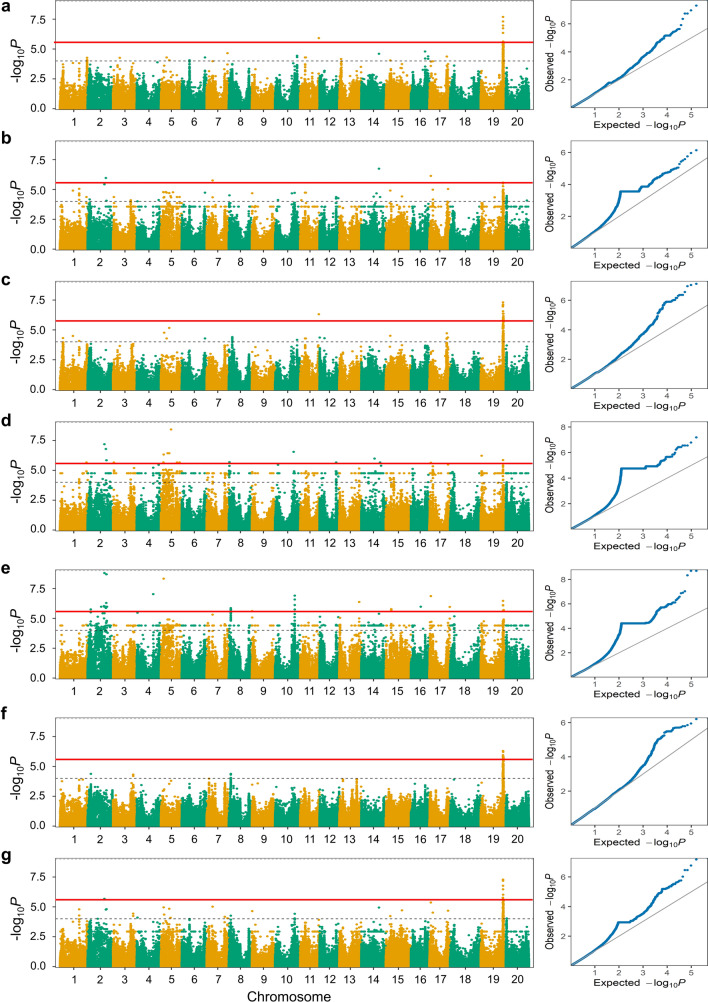
GWAS signals for plant height evaluated across six environments. Manhattan plot and quantile–quantile (Q–Q) plot for the GWAS for PH evaluated at **a** Nanjing_2011 (E1), **b** Nantong_2011 (E2), **c** Yangzhou_2011 (E3), **d** Nanjing_2012 (E4), **e** Nantong_2012 (E5), and **f** Yangzhou_2012 (E6). The red lines on the *Y*-axis designate the significance threshold (− log_10_ *P* < 5.47). The numbers on the *X*-axis represent soybean chromosomes

**Fig. 2:**
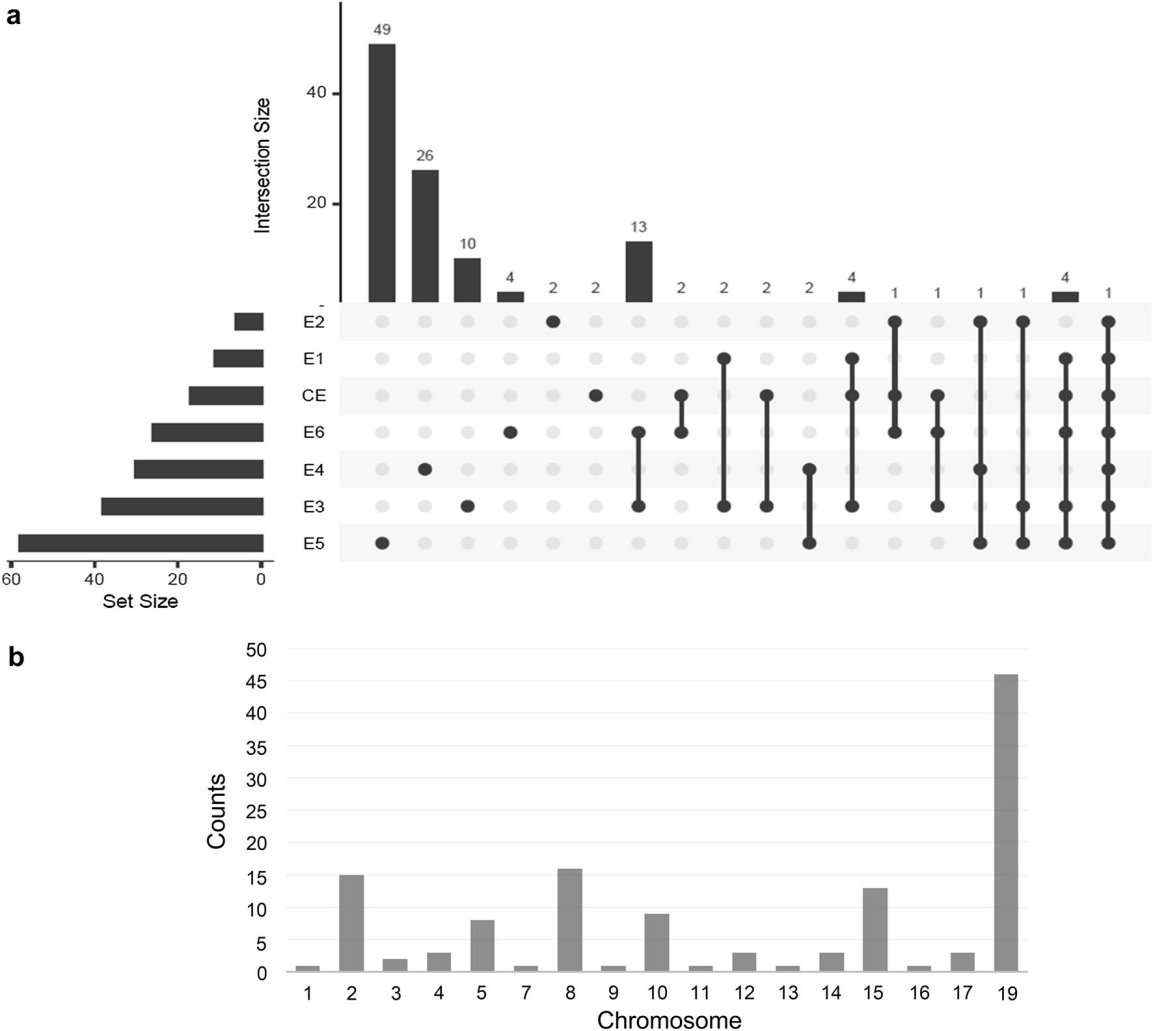
Intersection of significant QTLs and their frequency across soybean chromosomes. **a** UpSet plot showing the intersection of significant QTLs across six different environment (E1, E2, E3, E4, E5, and E6) and combined environment (CE). **b** Frequency of QTLs across soybean chromosomes

A total of 128 SNPs were found to be significantly associated with PH across six environments and the combined environment, based on GWAS results (Fig. 1; Supplementary Table 6). The distribution of markers across soybean chromosomes and their intersections based on the individual and combined environments are presented in Fig. 2 as UpSet plot (Lex et al. 2014). The UpSet plots provides a convenient alternative to visualize the data sets by frequency. In Fig. 2a, the horizontal bar graph on the left-hand side of the UpSet plot showed the size of each set, i.e., total number of significant SNPs identified in each individual environment and the combined environment. For example, the horizontal bar linked to E5 environment indicated the presence of 58 significant SNPs identified in this environment. Similarly, the horizontal bars corresponding to other five individual environments (E1, E2, E3, E4, and E6) and the combined environment showed the number of significant SNPs identified in each of these environments (Fig. 2a). Furthermore, the vertical bars on the top indicate the individual set participation sizes (Fig. 2a). For instance, the vertical bar on the left most side shows that out of the 58 significant SNPs identified in E5 environment, 49 SNPs were specific for E5 environment, two SNPs were detected in E4 and E5, one SNP in E2, E4, and E5; one SNP in E2, E3, and E5; four SNPs in E1, E3, E5, E6, and CE; and one SNP was detected in E1, E2, E3, E4, E5, E6, and CE (Fig. 2a). In Fig. 2b, each vertical bar reveals the number of significant SNPs associated with PH identified on each chromosome. Notably, three significant SNPs were consistently identified in three or more environments (Fig. 2; Table 4). These included a SNP (AX-93897200) on Chr.19, which was detected in all six environments and the combined environment, a SNP (AX-93958260) on Chr.02 and a SNP (AX-94154834) on Chr.17 that were detected in three different environments. The regions within ~ 130 kb interval [based on the LD decay previously reported for the same population by using NJAU 355 K SoySNP assay (Wang et al. 2016)] flanking the significant SNPs (AX-93958260, AX-94154834, and AX-93897200) present on Chr.02, Chr.17, and Chr.19, were referred as *qPH2*, *qPH17* and *qPH19*, respectively. These QTLs/genomic regions represented stable genetic elements regulating PH in soybean.

**Table 4:** Stable QTLs/genomic regions associated with plant height consistently identified in at least three environments

QTL	Chromosome	Representative SNP^a^	Position (bp)	Number of significant SNPs	Environments	Related QTL	References
*qPH2*	Chr.02	AX-93958260	36,907,407	3	E2, E3, E5	*Plant height* 26–9	Sun et al. (2006)
*qPH17*	Chr.17	AX-94154834	2,929,975	1	E2, E4, E5	No related QTL	Not available
*qPH19*	Chr.19	AX-93897200	44,950,887	5	E1, E2, E3, E4, E5, E6, CE	*Plant height* 6–1; *Plant height* 10–4	Lark et al. (1995); Orf et al. (1999)

^a^The representative SNP with the minimum *P*-value; *CE* combined environment

### Superior haplotypes for PH identified on soybean Chr. 19

**Fig. 3:**
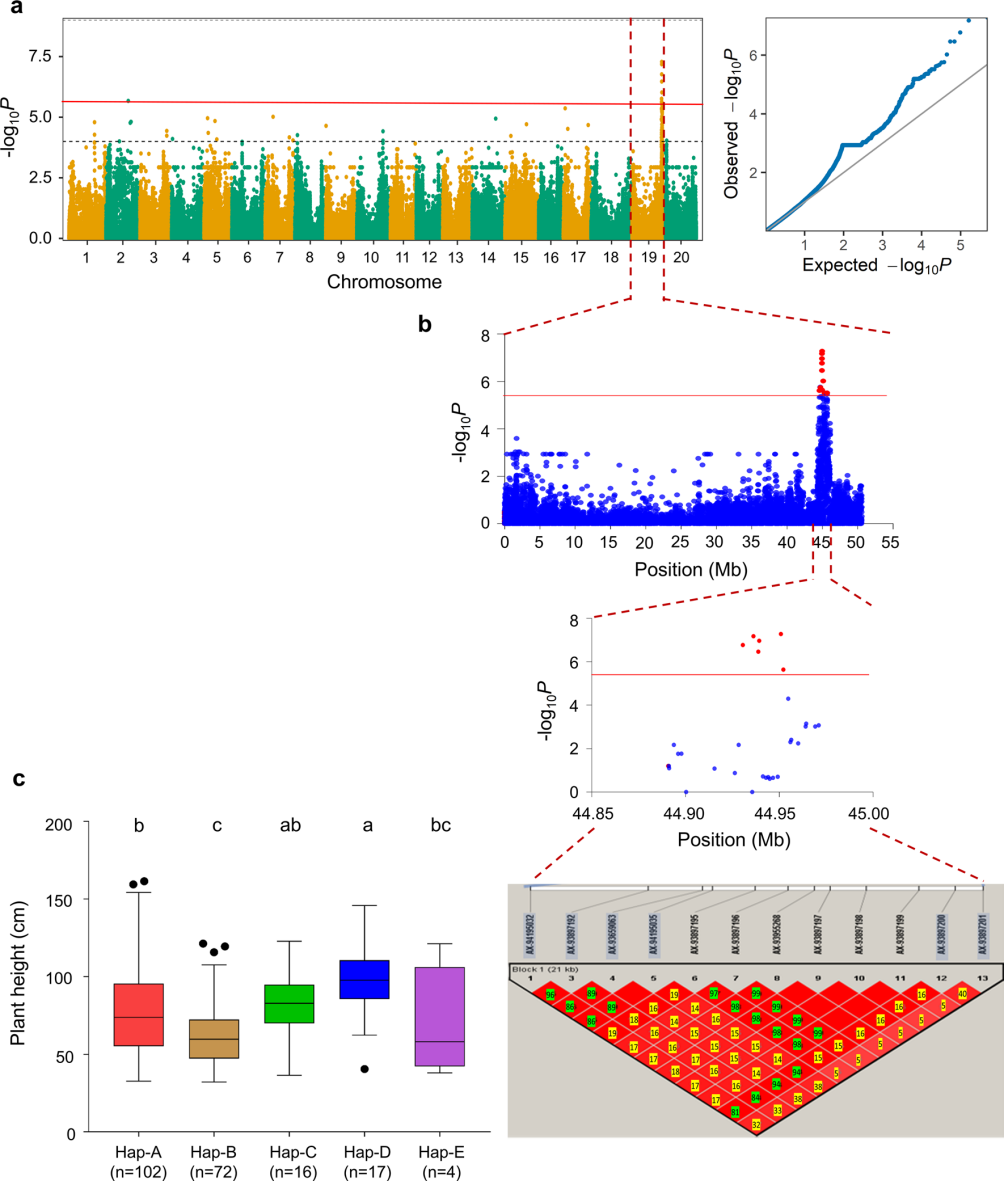
GWAS signal, haplotype block analysis of *qPH19*, and haplotype effect for plant height. **a** Manhattan plot and Q-Q plot for the GWAS for PH analyzed in the combined environment. Red line represents the Bonferroni correction threshold. **b** GWAS signal for PH obtained on Chr.19 and pairwise LD analysis. The pairwise LD diagram for significant variants (*P* < 3.42 × 10^–6^) is displayed. **c** The boxplot for the haplotypes based on the predicted values of PH in the combined environment (CE). The genotypes were grouped and pairwise comparisons were conducted using Turkey’s HSD Test at *P* < 0.05. The boxes with a common alphabet indicate no significant difference in PH. Number of accessions (*n*) in each sub-class is represented on top of each box

The genomic region flanking three stable significant SNPs (AX-93958260, AX-94154834, and AX-93897200) within the LD decay of ± 130 kb was subjected to haplotype analysis. The results showed that only significant markers identified within the LD decay flanking the SNP AX-93897200 (detected in all six environments and the combined environment) on Chr.19 formed a haplotype block. No haplotype block was detected for other two significant SNPs within the referenced LD decay (± 130 kb) on Chr.02 and Chr.17. Hence, the SNP (AX-93897200) along with 11 other SNPs (AX-94195032, AX-93897192, AX-93659063, AX-94195035, AX-93897195, AX-93897196, AX-93955268, AX-93897197, AX-93897198, AX-93897199, and AX-93897201) were found to be in strong LD and formed a single haplotype block (Fig. 3). The length of this haplotype block was less than 0.15 Mb. These 12 SNPs within the haplotype block represented five haplotypes across 211 accessions of the soybean natural population. Notably, these haplotypes classified the genotypes of the studied soybean population into five groups. The PH of the genotypes varied significantly across the five haplotype groups. For instance, Haplotype-A (Hap-A) formed the largest group (*n* = 102), followed by Hap-B (*n* = 72), Hap-C (*n* = 16), Hap-D (*n* = 17), and Hap-E (*n* = 04) (Fig. 3c). The nucleotide variation detected for these haplotypes is represented in Supplementary Fig. 2.

The differences in PH based on different haplotype alleles identified within the haplotype block on Chr.19 in soybean GWAS population varied across environments. However, soybean accessions with Hap-D appeared to be the tallest across environments, those with Hap-E and Hap-B had a dwarf phenotype, and accessions with Hap-A and Hap-C were of intermediate height (Fig. 3c; Supplementary Fig. 3).

### Identification of candidate genes within three QTL intervals

**Fig. 4:**
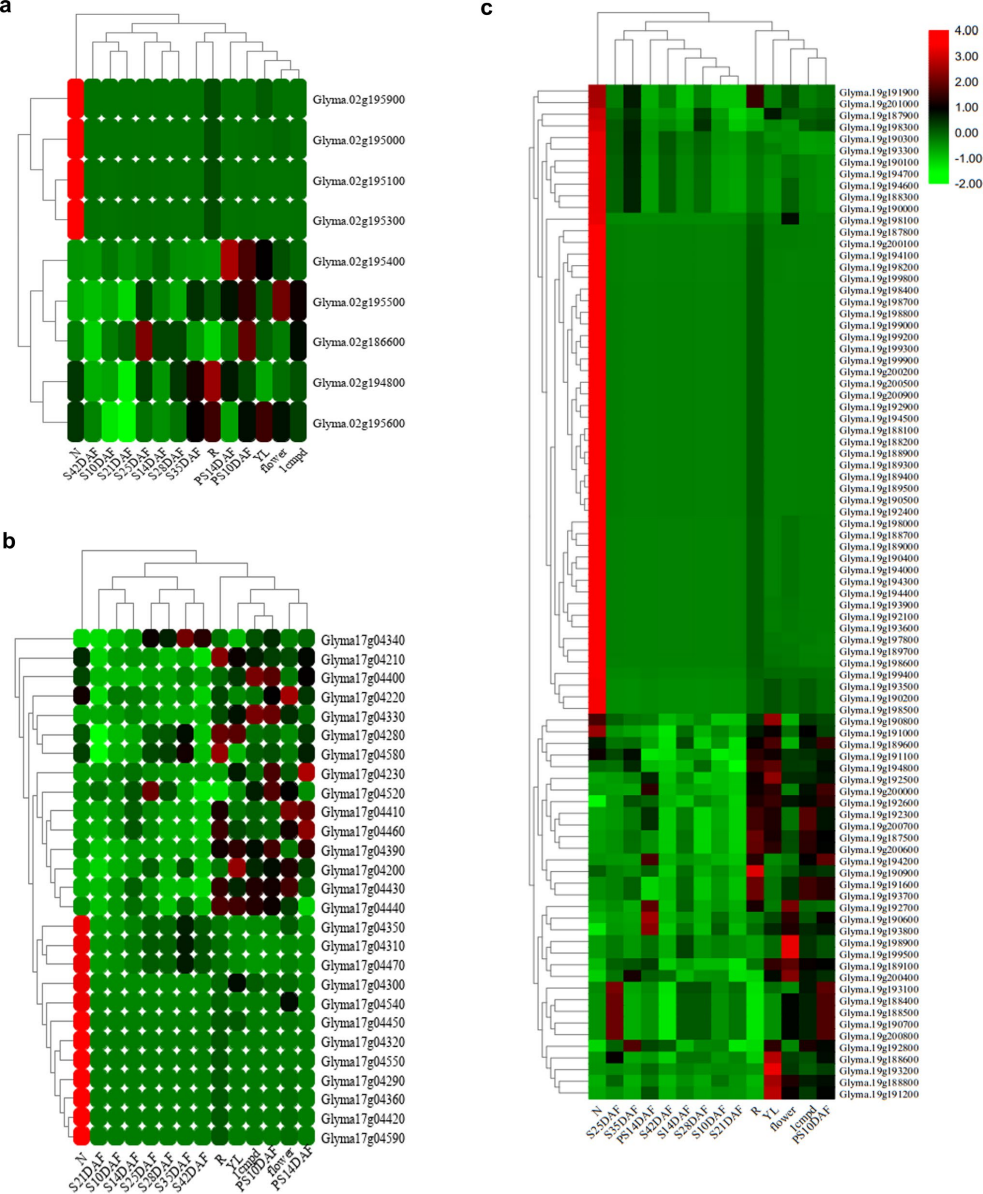
Heat map exhibiting the expression profiles of candidate genes underlying the target QTL intervals. The heat map represents the expression profiles of 124 candidate genes including 9, 27, and 88 genes present on Chr.02, Chr.17, and Chr.19 underlying three QTLs, **a** *qPH2*, **b** *qPH17,* and **c** *qPH19*. Heat map was generated using the RNA-seq data retrieved from online dataset at SoyBase. *N*, nodule; *S10DAF*, seed at 10 days after flowering; *S14DAF*, seed at 14 days after flowering; *S21DAF*, seed at 21 days after flowering; *S25DAF*, seed at 25 days after flowering; *S28DAF*, seed at 28 days after flowering; *S35DAF*, seed at 35 days after flowering; *S42DAF*, seed at 42 days after flowering; *PS10DAF*, pod shell at 10 days after flowering; *PS14DAF*, pod shell at 14 days after flowering; *YL*, young leaf; *1cmpd*, 1 cm of pod; *flower*, flower; and *R*, root

A total of 169 genes were identified within the physical genomic interval of all three major QTLs (*qPH2*, *qPH17* and *qPH19*)*,* which consisted of 18, 35, and 116 genes, respectively (Supplementary Table 7). Of the 169 genes detected, the RNA-seq data for only 124 genes were available online at SoyBase (https://www.soybase.org/) for 14 soybean tissues across different stages of growth and development (Supplementary Table 4). Expression pattern of the genes obtained from the RNA-seq data is represented in the form of a heatmap in Fig. 4. Furthermore, based on in silico analysis of gene expression data and gene annotations, we defined a total of 22 candidate genes underlying three stable genomic regions. This includes two genes underlying *qPH2*, five genes underlying *qPH17,* and 15 genes underlying *qPH19* (Table 5). The selection of these candidate genes was based on gene function annotation (i.e., genes regulating cell division, meristem growth, plant growth hormone biosynthesis and signaling, cell elongation, and transition from vegetative to reproductive phase), literature studies, and gene expression profiles (Table 5; Supplementary Table 4). These 22 genes can be considered as putative candidates controlling PH. However, further functional validation of these genes is needed to determine their exact role in regulating PH in soybean.

**Table 5:** Potential candidate genes underlying the three QTLs (*qPH2*, *qPH17,* and *qPH19*) and their gene annotation

Serial no.	Gene ID (Wm82.a4.v1)	Gene ID (Wm82.a2.v1)	Biological functions
1	Glyma02g35210	Glyma.02g195300	Regulation of stomatal movement; response to auxin stimulus; response to ethylene stimulus
2	Glyma02g35350	Glyma.02g195900	MAPK cascade; intracellular signal transduction; protein ubiquitination
3	Glyma17g04210	Glyma.17g037900	Aerobic respiration; cell redox homeostasis; response to fructose stimulus; response to glucose stimulus; response to sucrose stimulus
4	Glyma17g04300	Glyma.17g038700	Cytokinesis; cytokinesis by cell plate formation; microtubule-based movement; nuclear division; nucleolus organization; regulation of DNA replication; regulation of G2/M transition of mitotic cell cycle
5	Glyma17g04340	Glyma.17g039100	S-adenosylmethionine biosynthetic process; lignin biosynthetic process; methionine metabolic process
6	Glyma17g04350	Glyma.17g039200	Auxin polar transport; regulation of anion channel activity; root development
7	Glyma17g04360	Glyma.17g039300	Auxin polar transport; regulation of anion channel activity; root development
8	Glyma19g37380	Glyma.19g189700	Bract formation; floral meristem determinacy; leaf morphogenesis; positive regulation of transcription, regulation of timing of transition from vegetative to reproductive phase; regulation of transcription
9	Glyma19g37410	Glyma.19g190000	Determination of bilateral symmetry; meristem initiation; polarity specification of adaxial/abaxial axis; regulation of meristem growth; regulation of transcription
10	Glyma19g37530	Glyma.19g191100	Embryo development ending in seed dormancy; lipid storage; meristem structural organization; photomorphogenesis; photoperiodism, flowering; regulation of flower development; seed germination; vegetative to reproductive phase transition of meristem
11	Glyma19g37570	Glyma.19g191600	Gibberellin biosynthetic process; regulation of post-embryonic root development; regulation of stem cell division; regulation of timing of transition from vegetative to reproductive phase; response to fructose stimulus; response to sucrose stimulus
12	Glyma19g37610	Glyma.19g191900	DNA recombination; chromatin silencing by small RNA; meiosis; meiotic chromosome segregation; regulation of telomere maintenance; sister chromatid cohesion; telomere maintenance in response to DNA damage
13	Glyma19g37660	Glyma.19g192300	Determination of bilateral symmetry; meristem initiation; negative regulation of biological process; negative regulation of organ growth; organ morphogenesis; polarity specification of adaxial/abaxial axis; regulation of flower development; regulation of meristem growth; regulation of transcription, DNA-dependent; xylem and phloem pattern formation
14	Glyma19g37680	Glyma.19g192500	Biological process; cell differentiation; tissue development
15	Glyma19g37750	Glyma.19g192800	Amylopectin biosynthetic process; carbohydrate metabolic process; plant-type cell wall organization; polysaccharide catabolic process; starch biosynthetic process; starch metabolic process
16	Glyma19g38730	Glyma.19g200100	Anther development; double-strand break repair via homologous recombination; embryo development; embryo sac development; establishment of meiotic sister chromatid cohesion; leaf phyllotactic patterning; meiotic chromosome segregation; mitotic sister chromatid segregation; root development; sister chromatid cohesion
17	Glyma19g38760	Glyma.19g200400	Biological process; vegetative to reproductive phase transition of meristem
18	Glyma19g38790	Glyma.19g200700	Embryo development ending in seed dormancy; mevalonate-independent pathway; ovule development; thylakoid membrane organization; vegetative to reproductive phase transition of meristem
19	Glyma19g37770	Glyma.19g193100	RNA processing; mitotic cell cycle
20	Glyma19g37870	Glyma.19g194100	Adaxial/abaxial axis specification; autophagy; leaf shaping; leaf vascular tissue pattern formation; meristem maintenance; regulation of meristem growth
21	Glyma19g37890	Glyma.19g194300	Negative regulation of cell aging; negative regulation of flower development; photoperiodism, flowering; protein targeting to vacuole; regulation of flower development; response to sucrose stimulus
22	Glyma19g37910	Glyma.19g194500	Embryo development ending in seed dormancy; gibberellic acid mediated signaling pathway; gibberellin biosynthetic process; lipid storage; meristem structural organization; negative regulation of seed germination; photomorphogenesis; seed germination; sugar mediated signaling pathway; vegetative to reproductive phase transition of meristem

## Discussion

Plant height is an important determinant of soybean yield and quality (Lu et al. 2016; Yin et al. 2017). Taller soybean cultivars/varieties are often subjected to lodging, which leads to considerable reduction in soybean production as well as deterioration of its quality (Cao et al. 2019). Hence, tailoring PH in soybean to avoid qualitative and quantitative losses has been a long-term goal of soybean breeders. Determining the genetic basis of PH is a prerequisite to develop soybean varieties with desirable plant architecture. Studies have documented the complex inheritance of PH, besides the trait being strongly influenced by *G* × *E* interactions (Lee et al. 2015). Therefore, the present study was undertaken to identify the genetic loci/QTLs, and superior haplotypes regulating PH in soybean by using GWAS and haplotype analysis.

In the present study, ANOVA results revealed a significant difference among 211 accessions for PH (*P* < 0.01). In accordance with previous studies (Zhang et al. 2004; Cao et al. 2019), our study confirmed that PH is significantly affected by *G*, *E*, and *G* × *E* interactions. This suggests the complex genetic nature of PH in soybean. Furthermore, the estimate of broad-sense heritability for PH was high (98.95%) in the soybean natural population across all six environments, which is consistent with previous studies (Cao et al. 2019). Higher broad-sense heritability reported for PH in this study suggests a high probability of achieving the same phenotypic performance for PH when grown in the same environment. In agreement with our findings, many authors have earlier reported similar results for PH in the soybean germplasm collection (Zhang et al. 2015; Chang et al. 2018; Xue et al. 2019).

To date, the genetic basis of PH in soybean has been documented by multiple research groups (Orf et al. 1999; Chapman et al. 2003; Liu et al. 2013; Yin et al. 2017; Cao et al. 2019). However, the majority of previously detected QTLs have not been validated, which limit their deployment in MAB efforts. To this end, a more powerful and high-resolution mapping approach called the LD-based GWAS was used in the present study. Previous studies have demonstrated that GWAS is an effective strategy to determine the genetic basis of complex crop traits, such as PH, in a more precise and accurate manner (Kim et al. 2012; Cober and Morrison 2010). Hence, in the present study, we used GWAS for the identification of genomic regions associated with PH in soybean. We identified a total of 128 SNPs linked with PH across all the environments, which were found to be distributed on all chromosomes except Chr.06, Chr.18, and Chr.20. These results suggest the genome-wide distribution of loci/genes regulating PH in soybean and reveal the complex genetic architecture of this trait. In agreement with our results, Zhang et al. (2015) reported the distribution of significant MTAs for PH on 15 different chromosomes of soybean. Similar results have also been reported by other researchers (Fang et al. 2017, 2020; Chang et al. 2018). Further, the number of significant SNPs linked to PH was found to vary considerably across six different environments. For example, the highest number of SNPs were identified in E5, whereas the lowest number of SNPs were detected in E2. These findings suggest some effect of environment on the regulation of PH in soybean, which is in accordance with previous reports (Xue et al. 2019; Fang et al. 2020).

Importantly, three significant SNPs were consistently identified across multiple environments on Chr.02, Chr.17, and Chr.19. The genomic regions (~ 130 kb) flanking significant SNPs were referred as QTLs related to PH based on the LD decay. These QTLs/genomic regions represent stable genetic elements regulating PH in soybean. The QTL on Chr.02 (*Plant height *26–9) associated with PH has been previously reported in the genomic region between 15,993,654 and 41,032,570 bp by Sun et al. (2006), and the genomic region underlying *qPH2* was found to lie in the same physical interval. Therefore, *qPH2* might be the same as *Plant height* 26–9, as earlier reported by Sun et al. (2006). However, when compared to *Plant height* 26–9, the physical interval of *qPH2* was considerably narrowed down in the present study. Furthermore, *qPH19* identified in the present study was found to co-locate with two previously identified QTLs viz., *Plant height* 6–1 (44,862,405–45,270,324 bp) and *Plant height* 10–4 (44,862,405- 45,270,324 bp) on Chr.19 (Lark et al. 1995; Orf et al. 1999). However, there is no QTL identified to date that falls within the physical interval of *qPH17*. Hence, *qPH17* can be regarded as a novel QTL identified in the current study. The physical intervals of *qPH2* and *qPH19* were considerably reduced in the present study. This may be because the earlier reported QTLs (*Plant height* 26–9, *Plant height* 6–1, and *Plant height* 10–4) were identified via linkage mapping by using low-density markers such as simple sequence repeat and restriction fragment length polymorphism, possessing a relatively low genomic resolution. These marker systems have low selection accuracy, which has been the biggest obstacle for their deployment in the MAB programs. QTL mapping involves the use of bi-parental mapping populations, which are associated with poor resolution in the detection of target loci regulating traits of interest (Kraakman et al. 2004). To this end, GWAS has emerged as a powerful approach using the ancestral recombination events and has high-resolution in the detection of MTAs. Therefore, high resolution of GWAS in the identification of the stable QTLs across multiple environments will facilitate their effective utilization in MAB programs for breeding soybean varieties with desirable PH.

In the practical breeding programs, the identification of potential markers/candidate genes for the trait of interest underlying a particular genomic region is the ultimate objective (Ganie and Ahammed 2021; Ganie et al. 2021). To date, minimal efforts have been made to identify candidate genes regulating PH in soybean (Liu et al. 2013; Lee et al. 2015; Cao et al. 2017), and only few genes have been characterized (Liu et al. 2010; Ping et al. 2014; Zhang et al. 2018). Hence, based on literature research and gene annotations, we defined 22 candidate genes underlying *qPH2*, *qPH17*, and *qPH19* that may possibly regulate PH. For example, *Dt1* (*Glyma.*19G194300) gene lying within the physical interval of *qPH19* was considered as the candidate gene. The same locus on Chr.19 underlying the *Dt1* locus was also identified previously (Sonah et al. 2015; Kato et al. 2019). Liu et al. (2010) observed regulation of the growth habits and PH in soybean by *Dt1*. In addition to the growth habit genes, maturity and flowering time genes also have a major impact on PH (Cober and Morrison 2010; Xia et al. 2012; Zhang et al. 2015; Cao et al. 2017). Gene function related to growth hormones, mitosis, cell division, cell elongation or functions that are directly or indirectly related to the vegetative growth are involved in regulating PH. To this end, a total of 169 gene models were found underlying the physical intervals of three stable QTLs, and 22 genes (including *Dt1*) were defined as putative candidates controlling PH by taking into consideration their annotations and gene expression patterns. However, further verification and functional validation of these genes are required to precisely pinpoint their role in the regulation of PH in soybean.

Recently, haplotype-based breeding has emerged as a promising approach to develop custom-designed crop varieties (Varshney et al. 2021). However, this breeding approach needs detection of superior haplotypes for their ultimate use in breeding programs. Haplotype analysis allows plant breeders to utilize the genetic variation underlying key genes/loci to the greatest extent. For example, in soybean, a candidate gene regulating salinity tolerance (*GmCHX1*) was subjected to haplotype analysis. Among various haplotypes identified for *GmCHX1*, *SV-2* was found to provide maximum salinity tolerance (Patil et al. 2016). Moreover, Wang et al. (2017) identified superior haplotypes for grain quality traits (cooking and eating quality traits) in rice. Sinha et al. (2020) performed haplotype analysis of five genes controlling drought tolerance in pigeonpea. In a given genomic region, many factors regulate haplotype variation, such as mutation, recombination rates, and selection (Zaitlen et al. 2005). Hence, it is a prerequisite to include heterozygous haplotypes to capture maximum haplotypic variation for the analysis of haplotype diversity (Sinha et al. 2020). In our study, the genomic region flanking the most stable significant SNP (AX-93897200) that was in strong LD with 11 significant SNPs formed a single haplotype block. Five haplotypes (Hap-A, Hap-B, Hap-C, Hap-D, and Hap-E) were detected in this haplotype block. The results showed that five haplotypes identified in the block on Chr.19 regulate PH in soybean from dwarf ➝ intermediate ➝ tall. However, our results also reported that these haplotype alleles showed significant environment interaction across six different environments in the regulation of PH. This suggests the presence of haplotype × environment interaction, which needs to be fixed before the utilization of superior haplotype(s) in crop improvement.

A previous study has documented only two haplotypes underlying the *Dt1* locus viz., Gm19_Hap42b and Gm19_Hap42a regulating two different phenotypes of PH (Contreras-Soto et al. 2017). This haplotype block showed only two haplotypes (biallelic), and the significant SNP within this region was also biallelic. Therefore, this study showed no difference between the uses of the haplotype or SNP markers within the target region for MAB. The main objective behind the deployment of haplotype markers is that they are multi-allelic and thus provide better opportunity to modify the plant trait. For example, in the present study we identified five different haplotypes for PH underlying the *Dt1* locus viz., Hap-A, Hap-B, Hap-C, Hap-D, and Hap-E which regulated five different phenotype classes of PH in soybean. This provides a greater possibility to modify PH in soybean. Therefore, the incorporation of these haplotypes via haplotype-based breeding in soybean breeding programs will be very useful to tailor plant architecture and improve yield and quality.

## Conclusion

The present study identified 128 significant SNPs and three stable QTLs/genomic regions (*qPH2*, *qPH17,* and *qPH19*) associated with PH, which were consistently detected in at least three environments. Out of these three QTLs, the QTLs co-localizing with *qPH2* and *qPH19* have been previously detected by other researchers. However, no QTL was found to be associated with *qPH17*, implying this locus to be a novel genomic region regulating PH. A total of 22 candidate genes underlying these three QTL regions were also prioritized. The putative candidates defined within the intervals of *qPH2*, *qPH17,* and *qPH19* need further functional validation before they can be used in soybean breeding programs. The haplotype analysis detected five haplotypes (Hap-A, Hap-B, Hap-C, Hap-D, and Hap-E) within the stable genomic region of Chr.19, which regulated PH ranging from dwarf ➝ intermediate ➝ tall. The superior haplotypes identified in the Chinese soybean germplasm will serve as a potential resource for haplotype-based breeding of PH in soybean.

## Supplementary Information

Below is the link to the electronic supplementary material.

Supplementary file1 (DOC 1587 KB)

Supplementary file2 (XLSX 22 KB)

Supplementary file3 (XLSX 103 KB)

Supplementary file4 (CSV 195568 KB)

Supplementary file5 (XLSX 28 KB)

Supplementary file6 (XLSX 9 KB)

Supplementary file7 (XLSX 21 KB)

Supplementary file8 (XLSX 65 KB)

Supplementary file9 (DOCX 23 KB)

## Data Availability

All relevant data can be found within the manuscript and in Supplementary Material.

## Code availability

The scripts used for performing different analysis in the present study are provided in Supplementary File 1.
