# Supplementary material for: Identification of superior haplotypes in a diverse natural population for breeding desirable plant height in soybean
Source: Theor Appl Genet. 2022 May 31;135(7):2407–22. doi: 10.1007/s00122-022-04120-0 (PMC9271120; doi:10.1007/s00122-022-04120-0)
Supplement: Supplementary file 1 — Supplementary file1 (DOC 1587 KB) [file 122_2022_4120_MOESM1_ESM.doc]

**Identification of superior haplotypes in a diverse natural population for breeding desirable plant height in soybean**

Javaid Akhter Bhat^1,2*^, Benjamin Karikari^3^, Kehinde Adewole Adeboye^4^,

Showkat Ahmad Ganie^5^, Rutwik Barmukh^6^, Dezhou Hu^1^，Rajeev K. Varshney^6,7*^, Deyue Yu^1*^

^1^National Center for Soybean Improvement, State Key Laboratory of Crop Genetics and Germplasm Enhancement, Nanjing Agricultural University, 210095 Nanjing, China

^2^International Genome Center, Jiangsu University, Zhenjiang-212013, China

^3^Department of Crop Science, Faculty of Agriculture, Food and Consumer Sciences, University for Development Studies, Tamale Ghana

^4^Department of Agricultural Technology, Ekiti State Polytechnic, P. M. B. 1101, Isan, Nigeria

^5^Department of Plant Science and Landscape Architecture, University of Connecticut, Storrs, United States of America (USA)

^6^Center of Excellence in Genomics & Systems Biology, International Crops Research Institute for the Semi-Arid Tropics (ICRISAT), Hyderabad, 502324, India

^7^State Agricultural Biotechnology Centre, Crop Research Innovation Centre, Food Futures Institute, Murdoch University, Murdoch, Western Australia, Australia

**^*^Correspondence:**

[javid.akhter69@gmail.com](mailto:javid.akhter69@gmail.com) (Javaid Akhter Bhat);

[rajeev.varshney@murdoch.edu.au](mailto:rajeev.varshney@murdoch.edu.au) (Rajeev K. Varshney);

[dyyu@njau.edu.cn](mailto:dyyu@njau.edu.cn) (Deyue Yu)

**Supplementary Figures**

**
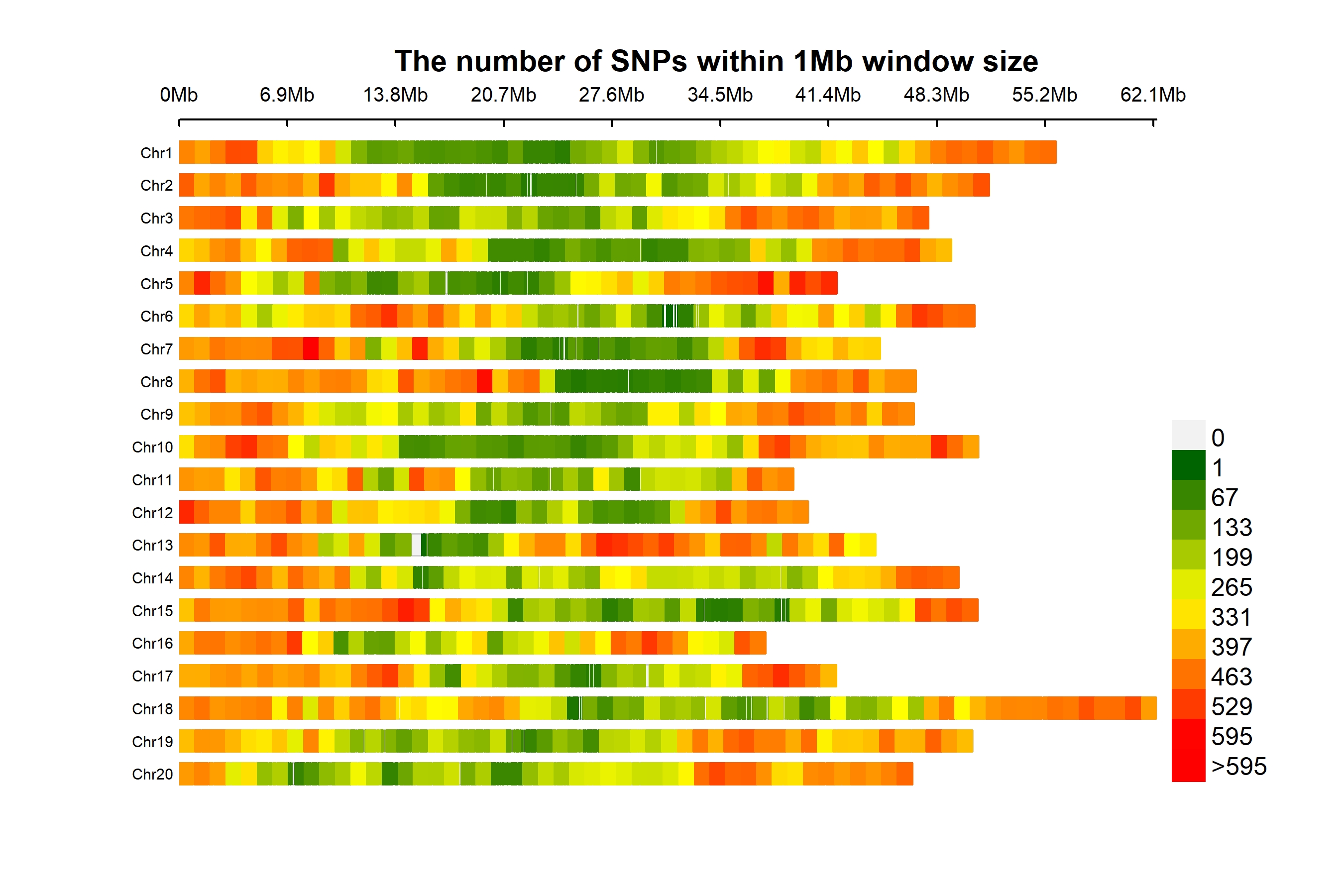
**

**Supplementary Figure 1: Distribution of 291,962 SNPs on 20 chromosomes of soybean.** The horizontal axis represents chromosome length (Mb); the vertical axis indicates chromosome number, and the different colors depict SNP density (the number of SNPs per window).


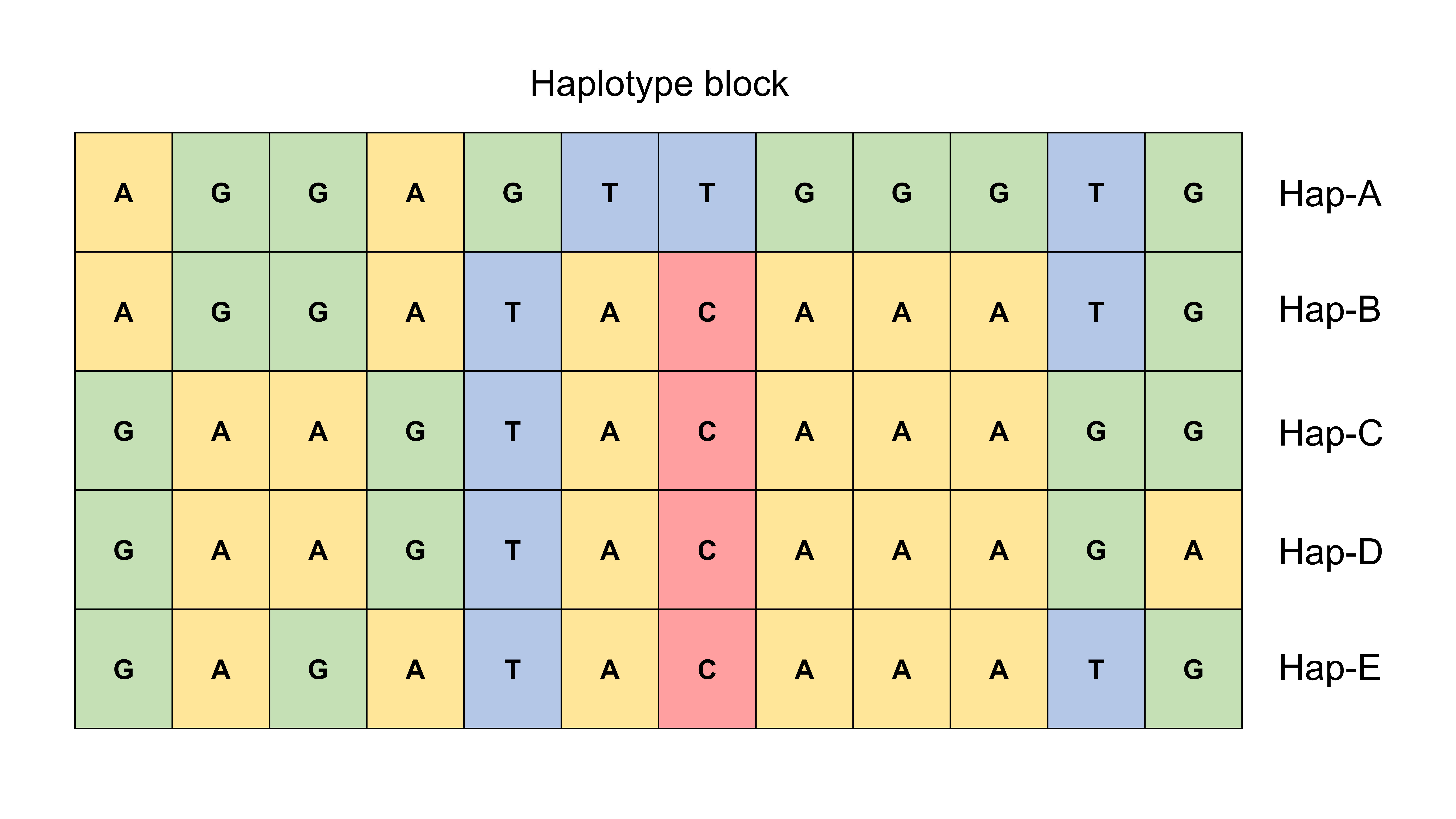


**Supplementary Figure 2: Nucleotide variation in the haplotype block on Chr.19**. Nucleotide variation across five haplotypes (Hap-A, Hap-B, Hap-C, Hap-D, and Hap-E) identified in the haplotype block on Chr.19 in a natural population containing 211 diverse soybean germplasm accessions.


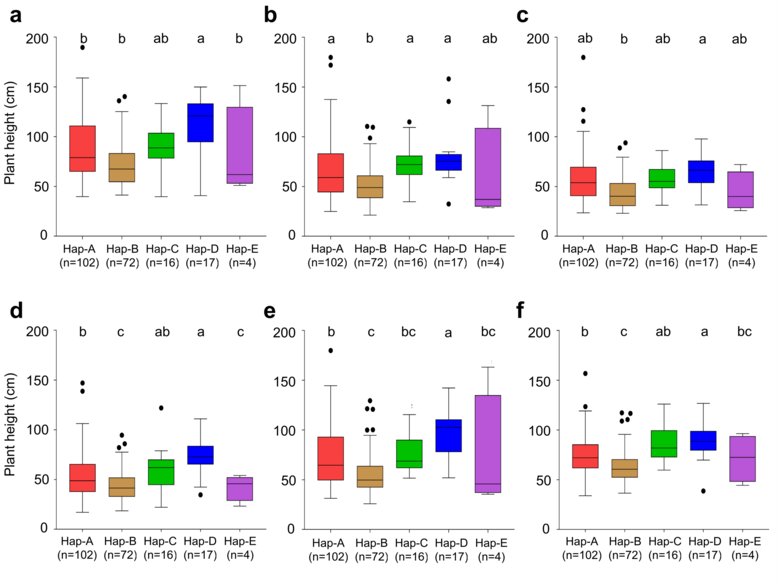


**Supplementary Figure 3: Effect of haplotypes on soybean plant height.** The figure depicts the effect of haplotypes on soybean plant height evaluated across (a) E1, (b) E2, (c) E3, (d) E4, (e) E5, and (f) E6 environments.
