# Supplementary material for: Identification of superior haplotypes in a diverse natural population for breeding desirable plant height in soybean
Source: Theor Appl Genet. 2022 May 31;135(7):2407–22. doi: 10.1007/s00122-022-04120-0 (PMC9271120; doi:10.1007/s00122-022-04120-0)
Supplement: Supplementary file 9 — Supplementary file9 (DOCX 23 KB) [file 122_2022_4120_MOESM9_ESM.docx]

**Identification of superior haplotypes in a diverse natural population for breeding desirable plant height in soybean**

Javaid Akhter Bhat^1,2*^, Benjamin Karikari^3^, Kehinde Adewole Adeboye^4^,

Showkat Ahmad Ganie^5^, Rutwik Barmukh^6^, Dezhou Hu^1^，Rajeev K. Varshney^6,7*^, Deyue Yu^1*^

^1^National Center for Soybean Improvement, State Key Laboratory of Crop Genetics and Germplasm Enhancement, Nanjing Agricultural University, 210095 Nanjing, China

^2^International Genome Center, Jiangsu University, Zhenjiang-212013, China

^3^Department of Crop Science, Faculty of Agriculture, Food and Consumer Sciences, University for Development Studies, Tamale Ghana

^4^Department of Agricultural Technology, Ekiti State Polytechnic, P. M. B. 1101, Isan, Nigeria

^5^Department of Plant Science and Landscape Architecture, University of Connecticut, Storrs, United States of America (USA)

^6^Center of Excellence in Genomics & Systems Biology, International Crops Research Institute for the Semi-Arid Tropics (ICRISAT), Hyderabad, 502324, India

^7^Murdoch’s Centre for Crop & Food Innovation, State Agricultural Biotechnology Centre, Food Futures Institute, Murdoch University, Murdoch, Western Australia, Australia

**^*^Correspondence:**

[javid.akhter69@gmail.com](mailto:javid.akhter69@gmail.com) (Javaid Akhter Bhat);

[rajeev.varshney@murdoch.edu.au](mailto:rajeev.varshney@murdoch.edu.au) (Rajeev K. Varshney);

[dyyu@njau.edu.cn](mailto:dyyu@njau.edu.cn) (Deyue Yu)

***Scripts used for performing different analysis in the present study***

**1. Compressed Mixed Linear Model (CMLM) for Genome Wide Association Study (GWAS) using GAPIT**

install.packages("BiocManager")

biocLite("multtest")

install.packages("gplots")

install.packages("LDheatmap")

install.packages("genetics")

install.packages("ape")

install.packages("EMMREML")

install.packages("Matrix")

install.packages("gtool")

install.packages("MASS")

install.packages("mvtnorm")

library(multtest)

library(gplots)

library(LDheatmap)

library(genetics)

library(ape)

library(EMMREML)

library(compiler) #this library is already installed in R

library("scatterplot3d")

source("http://zzlab.net/GAPIT/gapit_functions.txt")

source("http://zzlab.net/GAPIT/emma.txt")

setwd("D:\\myGAPIT")

myY <- read.table("mdp_traits.txt", head = TRUE)

myG <- read.table("mdp_genotype_test.hmp.txt" , head = FALSE)

#basic

myGAPIT <- GAPIT(

Y=myY,

G=myG,

PCA.total=3

)

#CMLM

myGAPIT <- GAPIT(

Y=myY,

G=myG,

PCA.total=3,

kinship.cluster=c("average", "complete", "ward"),

kinship.group=c("Mean", "Max"),

group.from=200,

group.to=1000000,

group.by=10

)

**2. Manhattan plot**

> CMplot(mydata,

type="p",

plot.type="m",

LOG10=TRUE,

threshold=NULL,

file="jpg",

memo="",

dpi=300,

file.output=TRUE,

verbose=TRUE,

width=14,

height=6,

chr.labels.angle=45)

# 'chr.labels.angle': adjust the angle of labels of x-axis (-90 < chr.labels.angle < 90).

**3.** **Quantile-Quantile (QQ) Plot**

> CMplot(mydata,

plot.type="q",

box=FALSE,

file="jpg",memo="",

dpi=300,

conf.int=TRUE,

conf.int.col=NULL,

threshold.col="red",

threshold.lty=2,

file.output=TRUE,verbose=TRUE,width=5,

height=5)

**4.** **Estimation of Variance components and BLUP values**

## Set Working Directory for Mac

setwd("/Users/heathermerk/Documents/TBRT2011")

## Set Working Director for PC

setwd("C:/your_working_directory")

## Read in Brix dataset

qualdat = read.csv("TBRTQuality.csv", header=T)

## Check to ensure data imported correctly

str(qualdat)

head(qualdat)

tail(qualdat)

#*For BLUP*

library(Matrix)

library(lme4)

data<- read.table("CE.txt",header=T)

data$line <- as.factor(data$line)

data$rep <- as.factor(data$rep)

data$env <- as.factor(data$env)

models <- lmer(sw~ (1|env) + (1|rep%in%env) + (1|line)+ (1|line:env),data=data,,control=lmerControl(check.nlev.gtr.1="ignore"))

a<- ranef(models)

BLUP<-a$line

write.table(BLUP,"CE_BLUP.txt",sep="\t")

## Calculate variance components

# requires lme4 package

library(lme4)

# Linear Model with random effects for variance components

brixvarcomp = lmer(BRIX~ (1|LINE) + (1|ENV) + (1|REP%in%ENV) + (1|LINE:ENV))

# Extract variance components

summary(brixvarcomp)

**6. Analysis of variance for haplotypes**

Boxplot with mean comparison

rm(list = ls())

#set working directory

setwd("C:/Users/user/Desktop/m_download")

inp.data1 <- read.table("PHHapdata.txt", header = T)

library(ggplot2)

library(agricolae)

library(dplyr)

### hap for combined environment

value_max = inp.data1 %>% group_by(HAP) %>% summarize(max_value = max(CE))

hsd=HSD.test(aov(CE~HAP, data=inp.data1), trt = "HAP", group = T)

hsd

sig.letters <- hsd$groups[order(row.names(hsd$groups)), ]

p <- ggplot(inp.data1, aes(x = HAP, y = CE))+

geom_boxplot(aes(fill= HAP))+

geom_text(data = value_max, aes(x=HAP, y = 0.1 + max_value, label = sig.letters$groups), vjust=0)+

stat_boxplot(geom = 'errorbar', width = 0.1)+

ggtitle("Haplotype Effect on PH in Combined Environment (CE)") + xlab("") + ylab(""); p

**7. Upset plot**

install.packages("UpSetR")

library(UpSetR)

dataph <- read.table("ph_mta.txt", header=TRUE)

#best figure for me

upset(dataph, nsets = 8, nintersects = 30, mb.ratio = c(0.5, 0.5), order.by = c("freq", "degree"), decreasing = c(TRUE,FALSE))

upset(dataph, sets = c("CE", "E1", "E2", "E3", "E4", "E5", "E6"), sets.bar.color = "#56B4E9",

order.by = "freq", empty.intersections = "on")

upset(dataph, attribute.plots=list(gridrows = 100, ncols = 1,

plots = list(list(plot=histogram, x="Chr",queries=T),

list(plot = scatter_plot, y = "AvgRating", x = "Watches", queries = T))),

sets = c("CE", "E1", "E2", "E3", "E4", "E5", "E6"),

queries = list(list(query = intersects, params = list("War"), active = T),

list(query = intersects, params = list("Noir"))))

upset(dataph,attribute.plots=list(gridrows=60,plots=list(list(plot=histogram, x="Chr")), ncols = 2))
